# The search for lunar mantle rocks exposed on the surface of the Moon

**DOI:** 10.1038/s41467-021-24626-3

**Published:** 2021-08-03

**Authors:** Daniel P. Moriarty, Nick Dygert, Sarah N. Valencia, Ryan N. Watkins, Noah E. Petro

**Affiliations:** 1 NASA Goddard Space Flight Center, Greenbelt, MD USA; 2 University of Maryland, College Park, MD USA; 3 Department of Earth and Planetary Sciences, University of Tennessee, Knoxville, TN USA; 4 Planetary Science Institute, Tucson, AZ USA

**Keywords:** Geochemistry, Inner planets, Mineralogy, Petrology, Rings and moons

## Abstract

The lunar surface is ancient and well-preserved, recording Solar System history and planetary evolution processes. Ancient basin-scale impacts excavated lunar mantle rocks, which are expected to remain present on the surface. Sampling these rocks would provide insight into fundamental planetary processes, including differentiation and magmatic evolution. There is contention among lunar scientists as to what lithologies make up the upper lunar mantle, and where they may have been exposed on the surface. We review dynamical models of lunar differentiation in the context of recent experiments and spacecraft data, assessing candidate lithologies, their distribution, and implications for lunar evolution.

## Abstract (web summary)

Vast, ancient impact basins scattered mantle materials across the lunar surface. We review lunar evolution models to identify candidate mantle lithologies, then assess orbital observations to evalutae the current distribution of these materials and implications for fundamental planetary processes.

## Introduction

Earth’s Moon is a keystone for understanding the formation and evolution of silicate bodies throughout the solar system and beyond. The lunar mantle is central to unraveling the history of the Moon, but our understanding of it is restricted by the limited scope of available observations. Currently, there are no definitively confirmed mantle materials in the lunar sample collection. Consequently, our understanding of the mantle (and its role in shaping surface features and interior properties) is derived primarily from geophysical observations, laboratory experiments, and petrological analyses of indirectly sourced products such as crustal and volcanic materials.

The past fifty years of lunar science and exploration have led to a general framework for the formation and evolution of the Moon and its mantle. Early lunar evolution was driven by two major episodes, which likely overlapped temporally: crystallization of a deep, global magma ocean and subsequent gravitational restructuring, a consequence of initially inverted density structures over several time and spatial scales within the crystallizing mantle.

Although the lunar science community largely agrees upon the first-order principles of these processes, there are critical gaps in our understanding of the sequence and timescale of magma ocean crystallization and overturn, and, subsequently, the final form, structure, and composition of the lunar crust and interior. The most direct way to advance our understanding of lunar evolution is through the identification and characterization of mantle rocks on the lunar surface.

What locations can future missions target to access such materials? The most promising options are impact basins, several of which are expected to have excavated completely through the lunar crust, exposing and melting mantle materials from depth. However, these basins exhibit notable diversity in composition and mineralogy on local and regional scales. To maximize the chances of successfully sampling the lunar mantle, it is essential to identify candidate rock types and their compositional properties.

Historically, scientists searching for lunar mantle materials with satellite data have made certain assumptions about what the Moon’s upper mantle might look like. Unfortunately, these assumptions do not always reflect a nuanced understanding of mantle evolution processes gained through sample analyses, laboratory experiments, and numerical models. It is essential that future analyses consider candidate mantle materials justified through this dynamic, multidisciplinary perspective, rather than relying on historical assumptions.

## Upper mantle compositions predicted by lunar evolution models

A fundamental starting point in searching for lunar mantle materials is to constrain the range of compositions and mineralogies that may have been present in the upper mantle, over depth ranges that may have been excavated and/or melted during the formation of large impact basins. It is important to acknowledge that lunar evolution was a dynamic process, and consequently the upper mantle exhibited dramatically different compositions at different times (and perhaps locations) throughout lunar history.

### Initial stratigraphy of the lunar interior

Lunar stratigraphy through time is rooted in an early, global, deep Lunar Magma Ocean (LMO). The LMO concept is perhaps the most significant discovery of the Apollo program, with far-reaching implications for our understanding of terrestrial planet formation and evolution. The LMO hypothesis was developed after distinctive mineralogic, petrologic and geochemical trends were noticed in Apollo 11 samples. Briefly, *Wood et al*.^1^ concluded that the thick, monomineralic lunar crust must have formed from fractional crystallization of a global melting event. This hypothesis was soon supported further by geochemical analyses^2,3^.

To better understand the LMO, a wide range of laboratory experiments and numerical simulations have been conducted, each attempting to address fundamental LMO systematics. LMO models depend on a challenging array of initial conditions, physical processes, and unknown variables including the LMO bulk composition, depth of melting, volatile content, and mode of crystallization. Although different LMO models vary in their particulars, there is a general pattern of similarities in model outcomes tied to observational constraints.

Generally speaking, the first mineral to crystallize from the cooling LMO was Mg-rich olivine, followed by orthopyroxenes with increasingly ferroan compositions. These minerals were denser than the coexisting liquid and sank to form a mafic cumulate pile at the base of the LMO. Throughout the crystallization process, the temperature, pressure, and composition of the residual melt continuously changed. As crystallization proceeded, the remaining liquid became progressively enriched in Ca and Fe until plagioclase and Ca,Fe-rich pyroxenes began to crystallize. While relatively dense pyroxenes sank, plagioclase crystals were less dense than the coexisting liquid and therefore rose through the liquid to form a flotation crust. Controls on crustal formation are explored further below.

As LMO crystallization neared completion, the final liquid dregs became increasingly enriched in Fe, Ti, and minor and trace elements incompatible in crystallizing minerals. Dense Ti-bearing ilmenite oxides and Fe-rich pyroxenes crystallized in increasing abundance, forming (IBCs). The incompatible minor and trace elements include Th, K, rare earth elements (REE), and P, collectively referred to as KREEP^4–6^. These elements were highly enriched in the final liquid dregs, referred to as “urKREEP”^6^. The final minerals to crystallize were KREEP-rich high-Ca pyroxenes and oxides (plus trace phases such as phosphates) in the uppermost mantle.

**Fig. 1:**
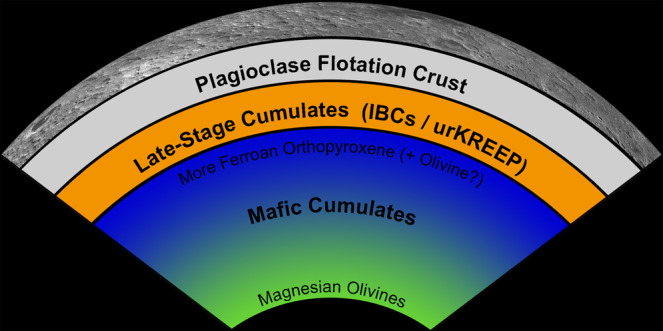
A generalized schematic overview the Lunar Magma Ocean cumulate stratigraphy. Because gravitational restructuring of the lunar mantle is probably initiated prior to complete magma ocean solidification, the actual structure of the lunar interior is not expected to exhibit this precise form at any point in lunar history. For example, late stage cumulates (including ilmenite-bearing cumulates (IBCs) and cumulates highly enriched in incompatible elements (urKREEP)) are thought to sink prior to complete solidification of the underlying mafic cumulates. Layer thicknesses are not to scale.

The details of LMO crystallization are still being explored. However, a generalized, widely accepted cumulate stratigraphy resulting from LMO crystallization considering a range of variables^7^ can be schematically conceptualized as three simplified units: Mafic Cumulates (initially stratified into a magnesian olivine-dominated lower mantle transitioning into a more ferroan orthopyroxene-dominated mineralogy at shallower depths), a Plagioclase Flotation Crust, and Dense Late-Stage Cumulates (including IBCs and urKREEP). This is illustrated in Fig. 1. For the purposes of this Perspective, we consider the lunar mantle to include all LMO products other than the flotation crust. Depending on the thickness of the crust and dense late-stage cumulate unit, the earliest lunar impact basins may have melted and ejected materials from several strata, including the crust, IBCs/urKREEP, and the uppermost mafic cumulates.

### Controls on the thickness of the plagioclase flotation crust

An important observational constraints on LMO crystallization models is the thickness of the lunar crust. A successful LMO model must be able to reproduce the ~40 km thick plagioclase flotation crust derived from orbital gravity measurements^8^. Crustal thickness is also relevant to exposure of mantle materials by impact basins. All else being equal, an impact basin formed within a region of thin crust is expected to excavate a larger volume of mantle materials from deeper stratigraphic layers, compared to a similar impact within a region of thicker crust.

The crustal thickness resulting from LMO crystallization is sensitive to a number of factors, mostly related to the quantity of plagioclase-forming elements (e.g., Al) in the magma ocean as it evolves. An important starting point is the lunar bulk composition. Differences in bulk composition are framed relative to the bulk composition of the Earth, specifically to what degree the Moon is enriched in refractory lithophile elements such as Al^9^. Estimates range from an Earth-like composition (Lunar Primitive Upper Mantle^9^) to a composition enriched in Al and other refractory lithophile elements by 50% (Taylor Whole Moon^10^). Because Al content is an important control on the crystallization of plagioclase, enrichment in Al results in a significantly thicker flotation crust^11,12^. Following similar logic, a deeper magma ocean would host more total Al than a shallower magma ocean, and therefore crustal thickness is sensitive to the initial depth of the LMO.

However, the relationship between Al enrichment and crustal thickness is not simply linear. For instance, crystallization of an Al-enriched LMO may result in the precipitation of garnet and spinel in the deep mantle^11^, reducing the quantity of Al available to form plagioclase later in LMO evolution.

Another factor affecting crustal thickness is the efficiency of separation between solids and melt in the crystallizing LMO. The most recent experiments investigating the viscosity of the lunar magma ocean liquid suggest that over the timescale of LMO crystallization, plagioclase flotation was very efficient, producing IBC largely free of plagioclase and a nearly pure plagioclase flotation crust^13^. However, if crystal flotation is inefficient, some plagioclase could remain trapped in the upper mantle. Similarly, it is possible that small pockets of Al-bearing melt become trapped within the matrix of early LMO mafic cumulates, reducing the amount of Al available to form plagioclase. Therefore, a higher volume of interstitial melt in mafic cumulates could reduce crustal thickness by several km^14^.

Crustal thickness is also sensitive to the presence of volatile elements in the LMO. The discovery of water in lunar pyroclastic glasses^15^ and remotely sensed at or near the lunar surface^16,17^ has established that the role of volatiles must be considered in lunar evolution models.

Given a fixed starting LMO composition, varying volatile content appears to affect the resulting crustal thickness. A recent set of experiments modeled LMO crystallization in the presence of 0, 0.5, and 1.0 wt.% water^7^. By adding water, plagioclase crystallization was both delayed and suppressed compared to their dry experiments, leading to a much thinner crust. However, more experiments and models are required to more definitively understand whether the presence of water in the LMO merely delays crystallization of plagioclase or significantly reduces the total mass of plagioclase that precipitates.

Plagioclase may not be the only major component of the primary crust. After 90% LMO crystallization, some experimental results indicate quartz is a stable phase^7,12,18^. This deviates from theoretical models, which do not predict the presence of quartz^19^. If present, quartz would contribute to a late-stage, low-density layer in the lower crust^7,12^, potentially resulting in a thicker overall crust than strictly allowed by Al content considerations alone. Although LMO quartz has not yet been detected, it may be present in deep crustal materials exposed by complex craters in the highlands. Quartz currently identified in the lunar sample collection is more consistent with post-LMO magmatic activity^20^.

### Evolution via gravitational restructuring

**Fig. 2:**
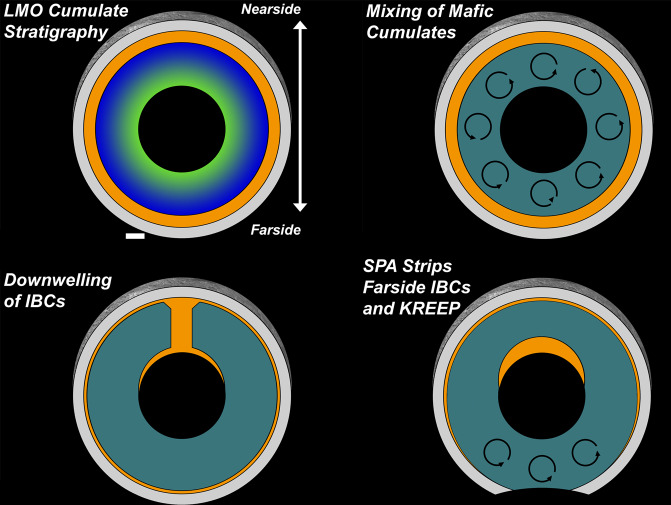
The evolution of the lunar interior through time. As mentioned in Fig. 1, the predicted Lunar Magma Ocean (LMO) cumulate stratigraphy (top panel) is never realized at any point in lunar history. Instead, dynamical models and experiments suggest that early mafic cumulates become mixed and dense ilmenite-bearing cumulates (IBCs) sink into the lower mantle before magma ocean solidification is complete (middle panels). Depending on viscosity contrasts between IBCs and the underlying mafic mantle, hemispherical downwelling may concentrate IBCs in the nearside mantle, which could be responsible for the dichotomy in thermal and geochemical evolution between the nearside and farside (however, ilmenite rheology estimates suggest this material would instead form a uniform, partially molten layer around the lunar core). Globally, some IBCs and incompatible elements (KREEP) are expected to remain in the upper mantle but may be stripped from the farside by South Pole – Aitken Basin (SPA) excavation and localized convection (bottom panel). Layer thicknesses are not to scale.

The initial cumulate stratigraphy is not expected to persist as the Moon continued to evolve. As LMO crystallization proceeded, the concentration of dense, relatively incompatible elements (such as Fe, Ti, and KREEP) continuously increased in the residual liquid, and thus in later-crystallizing cumulates (IBCs and urKREEP). Therefore, the initial LMO cumulate stratigraphy is thought to have been gravitationally unstable, with denser lithologies overlying less dense early magma ocean cumulates. Hence, it is expected that the lunar mantle underwent some degree of gravitational restructuring, affecting the compositional structure of the upper mantle. Several stages of this gravitational restructuring are represented schematically in Fig. 2.

The timescales, depth scales, and general nature of this restructuring are not fully understood, and implications for upper mantle compositions and magmatic geochemistry are more complex and nuanced than commonly assumed. For instance, gravitational restructuring may be strongly intertwined with LMO solidification processes, as restructuring is estimated to occur on shorter timescales. A schematic illustration of overturn processes is presented in Fig. 2, explored in more detail below.

How was the lunar mantle restructured, and what are the implications for the exposure of upper mantle materials by impact basins? Using dynamical scaling relationships and petrologic arguments, *Hess and Parmentier*^21^ defined the current cumulate mantle overturn paradigm by establishing a framework for estimating temporal and spatial scales for overturn. They evaluated the wavelength and sinking depth of IBC diapirs that formed during overturn, and found a strong dependence on IBC layer thickness and the viscosity contrast between IBC and underlying mafic cumulates. Importantly, they argued that sinking of the IBC starts before magma ocean solidification is completed. In their model, relatively small IBC diapirs sink into underlying mafic cumulates while magma ocean solidification progresses, forming a thickened (but poorly mixed) IBC layer with a relatively dilute IBC fraction. Depending on its viscosity, the thickened IBC layer may set the stage for a subsequent large-scale (perhaps hemispherical) overturn event. The possibility of hemispherical downwelling and implications for lunar evolution are explored in the following section.

Was gravitational restructuring indeed initiated before the LMO fully crystallized? Several recent numerical studies have explored the timing and efficiency of overturn to address this possibility. For model cases with reasonable IBC viscosities, the studies found that IBC downwelling would have occurred over timescales of millions to 10 s of Myr^22,23^. Because the lunar magma ocean solidified over 10s-100s of Myr based on sample ages and LMO crystallization models^14,23–27^, we may conclude that Hess and Parmentier^21^ were correct in arguing that the sinking of the IBC occurred during active magma ocean solidification. Following similar logic comparing timescales, it is expected that early mafic cumulates were themselves subject to gravitational restructuring and/or convection prior to complete solidification of the LMO^21,28^.

This has implications for the compositional structure of the entire lunar mantle. For magma oceans with crystallization timescales and viscosity contrasts such that gravitational restructuring occurs prior to complete solidification, the resulting post-overturn mantle is much more well-mixed than if the magma ocean fully crystallized prior to overturn^28^. For the Moon, this means that the early mafic cumulates (olivine and orthopyroxene) may become well-mixed rather than retaining the initial magnesian-to-ferroan cumulate stratigraphy^28^. While earlier models suggest the mafic cumulates undergo their own gravitational restructuring to form an inverted stratigraphy (ferroan-to-magnesian with decreasing depths)^21^, more recent analyses indicate that this scenario is only possible if LMO solidification was complete before gravitational restructuring^28^.

Since our best current estimates suggest that gravitational restructuring was initiated prior to complete LMO solidification, this suggests that mafic mantle cumulates became well-mixed early in lunar history^28^. If this is true, the upper mantle should at no point exhibit a monomineralic average composition after LMO solidification, even if LMO crystallization initially produced monomineralic cumulate layers. Instead, an ultramafic mixture of olivine and pyroxene with a range in magnesian-to-ferroan compositions is expected. This ultramafic assemblage therefore may be the most likely mantle product excavated by impact basins throughout lunar history. However, the efficiency and spatial scales of mafic cumulate mixing are not currently well-constrained. Therefore, it is possible that pockets of primordial monomineralic olivine or orthopyroxene could persist in the mantle^29^, even if the bulk mineralogy of the upper mantle is a mixture.

Alternatively, recent dynamical models suggest that a large proportion (>30%) of IBCs and urKREEP escape gravitational restructuring and remain in the shallow mantle throughout lunar history^23^. If this is indeed the case, the mixed ultramafic assemblage in the upper mantle may be overlain by late LMO cumulates: a Ti- and KREEP-bearing layer mineralogically dominated by ferroan pyroxenes and oxides. These late cumulates and liquid dregs may have also been excavated in larger proportions by the earliest lunar basins, if they formed prior to complete LMO solidification.

Historically, these gravitational restructuring processes have been referred to as “overturn”. Unfortunately, this term is often misunderstood to represent a discrete, large-scale event. In many cases, interpretations of remote sensing data are considered in the context of a “pre-“ or “post-overturn” lunar mantle. As described above, this is unlikely to be the case, according to our best understanding of LMO crystallization timescales and restructuring dynamics. Instead, mantle restructuring most likely occurs over multiple vertical scales before the complete solidification of the LMO. To better reflect the complexity of these processes, we recommend the use of the term “gravitational restructuring” rather than “overturn” in future publications.

### Origin of lunar asymmetries driven by mantle evolution

The Moon exhibits remarkable differences in crustal thickness, geochemistry, and volcanic activity between the nearside and farside. The nearside crust is thinner and more ferroan than the farside crust^8,30^, and the nearside hosts the overwhelming majority of volcanic deposits. Nearside basalts are associated with surficial Th and Ti anomalies^31^ in contrast to Th-free farside basalts^32^, implying an asymmetric distribution of KREEP and IBCs in the underlying mantle^33^. What is the origin of these asymmetries, and what are the implications for mantle materials exposed by lunar basins?

Several mechanisms for generating the observed hemispherical asymmetries have been proposed, associated with three primary drivers of lunar evolution: LMO crystallization, gravitational restructuring, and giant basin formation.

Some models predict that the LMO crystallized asymmetrically, with the farside crystallizing before the nearside. In this scenario, late-crystallizing elements (Ti, Th, KREEP) and more ferroan minerals would be concentrated on the nearside, consistent with the observed current-day compositional patterns. A consequence of this mechanism is that IBCs/KREEP would not be globally distributed after LMO crystallization, a testable prediction through analysis of ancient farside basin ejecta (considered below).

Alternatively, several models have explored whether the asymmetric IBC/KREEP distribution could be the result of gravitational restructuring^34,35^. To explain the hemispheric nature of these asymmetries, it is required that the driving restructuring mechanisms must have also been hemispheric or near-hemispheric. Rayleigh–Taylor stability analysis and numerical simulations suggest hemisphere-scale IBC downwelling may be possible if the viscosity contrast between the thickened IBC layer and underlying mafic cumulates is ~10,000^34,35^. Late LMO cumulates are weak^36,37^, and comparable degrees of viscosity contrast were observed in a recent study of xenoliths from a terrestrial mantle Rayleigh–Taylor instability^38^, but more experimental work on two-phase mixtures is needed to determine whether such a large viscosity contrast can also be expected for the lunar mantle. Even if downwelling is hemispheric, IBCs may form a uniform, stable layer enveloping the lunar core^37^. This could still be consistent with observed lunar asymmetries if the IBCs mixed to some degree with the mantle column during downwelling.

Because IBC downwelling is predicted to occur during LMO solidification^21,23,28^, this mechanism implies that IBCs/KREEP were not present in the upper mantle after solidification was complete. One exception to this is if IBC/KREEP downwelling was inefficient. If this was the case, >30% of IBCs and a larger fraction of KREEP could persist in the uppermost mantle^23^. These scenarios are testable through the evaluation of farside basin ejecta and volcanic emplacements.

The vast, ancient South Pole-Aitken Basin (SPA) on the lunar farside presents an ideal opportunity to test these hypotheses. Recent remote sensing analyses demonstrate that SPA ejected IBCs and KREEP-bearing rocks from the uppermost mantle^39^ (discussed in more detail below). This demonstrates that these materials were globally distributed in the LMO, not sequestered on the nearside by asymmetric crystallization.

Assuming the lunar lithosphere could not have preserved an impact structure the size of SPA before LMO solidification, this also suggests that IBC/KREEP downwelling was inefficient, since that IBC downwelling is thought to occur during LMO solidification^21,23,28^. However, this constraint might be relaxed if SPA did indeed form during the final stages of LMO crystallization.

From these observations, it is most likely that IBCs/KREEP were globally distributed during LMO crystallization and sequestered in the nearside mantle through hemispheric IBC downwelling. Inefficient downwelling left > ~30% of the initial IBC/KREEP content in the uppermost mantle^23^, which was later excavated by SPA formation.

What is the relationship between nearside KREEP sequestration and the KREEP-rich, volcanically prolific Procellarum region^40^? Conversely, another outstanding issue is the paucity of KREEP observed in farside volcanic emplacements^32^. These observations suggest that KREEP was not present in neither the farside mare source region nor upper mantle (where incompatible elements could be assimilated by ascending magmas). How could this be, if IBC/KREEP downwelling was globally inefficient?

It turns out that SPA formation itself could have influenced the distribution of IBCs/KREEP in the farside mantle. In addition to local removal of IBCs/KREEP by excavation, SPA formation could have induced vigorous regional mantle convection, which could have stripped IBCs and KREEP from a large swath of the farside mantle^41^. While global asymmetries in crustal thickness and composition could still be related to asymmetric LMO crystallization, asymmetries in IBC/KREEP distribution are best explained by hemispheric IBC downwelling in conjunction with regional geophysical implications of SPA formation.

## Evaluation of insights from recent observations

The lunar surface is pockmarked with impact craters and basins. Several of the largest basins are estimated to have excavated and melted mantle materials from beneath the ~40 km thick lunar crust^8,42,43^. However, there has not historically been consensus on what lithologies may comprise relevant upper mantle strata, and assumptions in observational papers often do not reflect the best current understanding of LMO petrology and gravitational restructuring dynamics. Several candidate lithologies have been commonly proposed based on observations and models: (1) dunite (monomineralic olivine), (2) low-Ca pyroxenite (monomineralic pyroxene), and (3) late LMO cumulates (IBCs/urKREEP). The following subsections evaluate the evidence for and against each of these cases.

### The case for an olivine-dominated upper mantle

Most lunar magma ocean models produce a dunite (monomineralic olivine) layer in the deep mantle. This dunite layer is less dense than later-crystallizing mafic and oxide cumulates, and may ascend via gravitational restructuring^21^. Early models have suggested a two-stage process to form a dunitic layer in the upper mantle: overturn of early mafic cumulates at the base of the LMO followed by dense overlying IBCs downwelling into the deep mantle^21^. However, more recent dynamical models suggest that the mafic cumulates become well-mixed^28^, excluding the possibility of widespread dunite in the upper mantle. However, a dunitic upper mantle would be expected if LMO mafic cumulates inverted without mixing^23^. Even if mixing did occur, localized pockets of primordial dunite could exist in the upper mantle^29^ if mixing was inefficient or progressed only on large spatial scales.

The occurrence of mantle-derived olivine in the lunar sample collection has been suggested, but remains unclear. Mantle-derived olivine has not been conclusively identified observed in the Apollo, Luna, or meteorite sample collections. Most olivine crystals in the sample collection exhibit shallow inferred crystallization pressures and evolved geochemical trends inconsistent with a deep, early mantle origin^44^. Instead, these samples are more consistent with post-LMO magmatic activity: differentiated plutons referred to collectively as the Mg-suite^45^. Intriguingly, Apollo 17 mare basalt sample 74275 contains xenoliths of dunite that are chemically, mineralogically, and texturally distinct from Mg-suite dunites^46,47^. While this hints at a possible mantle origin, recent analyses argue that these dunite xenoliths crystallized at shallower depths in the crust^44^.

Olivine-dominated materials have been identified via near-infrared reflectance spectroscopy in several locations across the lunar surface^48^. Could this olivine have originated from overturned mantle cumulates? Intriguingly, many of these olivine detections are associated with large impact basins modeled to excavate material from beneath the lunar crust^43^. This correlation is preliminarily suggestive of mantle exposures.

The nature of the olivine outcrops points to a more complex origin. The scale of each deposit is typically small. Rather than dominating basin structures, olivine presents as km-or-smaller-scale outcrops embedded within heterogeneous terrain, primarily feldspathic but often containing low-Ca pyroxene and spinel^48–51^. This heterogeneity is present in the olivine exposures themselves, as multiple olivine compositions have been observed within individual basins such as Moscoviense^49^. Furthermore, similar olivine compositions to these basin exposures have been observed in smaller craters (e.g. Copernicus^49^) that most likely expose crustal, not mantle, material^52^. Together, observations are more consistent with exposure of differentiated Mg-Suite plutons intruded into the lower crust, as opposed to the excavation of a thick, coherent, dunitic upper mantle^51^. The fact that the olivine-bearing basins are young (Nectarian and younger) and predominantly clustered on the nearside^43^ hints at a spatial and temporal dependence, again consistent with post-LMO magmatic petrogenesis^51^.

In light of mantle dynamic models indicating that the ultramafic mantle cumulates are likely well-mixed^28^, it is important to consider that olivine is difficult to detect in near-infrared reflectance spectra when mixed with pyroxenes^53^. However, the spatial scale of mixing is unclear, and it may be possible that km-scale pockets of monomineralic dunite may persist.

### The case for a pyroxene-bearing upper mantle

The case for an olivine-dominated upper mantle is further muddied by the compositional properties of the Moon’s largest impact basin. At over 2000 km in diameter, the South Pole-Aitken Basin (SPA) dominates the southern hemisphere of the lunar farside. Because of its immense scale, it is almost certain that SPA excavated and melted huge volumes of mantle materials^42,54^. However, there is little spectral evidence for abundant olivine within the basin. Instead, low-Ca pyroxene dominates spectral signatures in mantle-derived SPA impact melt^42,55–58^. This suggests that pyroxene was the dominant spectral component of the upper mantle at the time and place of the SPA-forming impact (although some olivine may also be present in the assemblage).

A pyroxene-bearing upper mantle is also supported by spectral analysis of other lunar impact basins. For instance, the melt sheet of the younger Crisium basin, which is estimated to include a significant mantle component, is spectrally dominated by low-Ca pyroxene^59^. However, it is unclear if lunar basin melt sheets reflect the bulk melt composition^60^ or differentiate into stratified layers with different compositions and mineralogies^61,62^. Avoiding this issue of differentiation, ultramafic, pyroxene-dominated outcrops around the Imbrium Basin have also been interpreted as mantle ejecta^63^.

Geophysical measurements further point to a pyroxene-bearing upper mantle. In particular, seismic velocities within the upper mantle, considered with the Moon’s mass and moment of inertia, appear to be most consistent with an orthopyroxene-bearing composition^64^. Of course, these seismic analyses are non-unique and are geographically limited to a small fraction of the lunar surface.

Did low-Ca pyroxenes in SPA and other basins originate in the upper mantle? If so, this is consistent with two possible scenarios. In accordance with our understanding of LMO crystallization and restructuring dynamics, the most likely scenario is that the pyroxene-bearing materials are actually ultramafic assemblages including an olivine component. This lithology is expected to be the result of early LMO mafic cumulates mixing during gravitational restructuring^28^. In this scenario, olivine is present but does not dominate the mineral assemblage, and therefore is difficult to detect using near-infrared reflectance spectroscopy.

If olivine is in fact not present, the pyroxene signature could be explained by several other scenarios. It is possible that the lack of olivine observed in SPA and Crisium impact melt could be a result of melt sheet differentiation^61,62^, though this would not explain the absence of olivine in SPA ejecta or undifferentiated melt^60^. Alternatively, a lack of olivine across SPA could point to partial mantle restructuring, where the early mafic cumulates at the base of the LMO did not themselves overturn, but were transported to the upper mantle due to downwelling of dense IBCs after the basin-forming impact. This seems unlikely, given our best understanding of LMO crystallization and overturn timescales and dynamics^21,28^.

One possible way to circumvent these challenging timescales is if SPA formed prior to complete LMO solidification. In this scenario, late-stage LMO products (IBCs / urKREEP dregs) would also remain in the uppermost mantle, immediately underlain by a monomineralic pyroxene-dominated unit. Could this be the case? In fact, some models of SPA melt sheet differentiation do suggest SPA formation melted materials matching the initial LMO cumulate stratigraphy^62^.

### The case for a KREEP- and IBC-bearing upper mantle

While it is true that SPA is dominated by pyroxene-bearing lithologies, the inferred upper mantle stratigraphy appears to be more complex than exclusively low-Ca pyroxene. Recent three-dimensional models of the SPA-forming impact suggest that mantle-derived ejecta is predominantly deposited and preserved in the NW quadrant of the basin^39,42^. The area modeled to exhibit the highest abundance of mantle-derived ejecta exhibits pyroxene compositions much higher in calcium and iron than the rest of SPA^39,57^. Furthermore, these high-Ca,Fe pyroxenes are strongly correlated with distinctive enhancements in thorium, iron, and titanium content^39^. This confluence of observations resembles the compositional properties of late LMO products—IBCs/urKREEP. This is evidence that the SPA-forming impact excavated very specific LMO mantle products either pre-downwelling or after incomplete downwelling^23^.

To first order, this confirms that KREEP was globally distributed in the mantle, as opposed to sequestered on the nearside. This is an important finding, as sequestration of KREEP has commonly been invoked to explain hemispherical differences in crustal thickness and volcanic activity^33,34,65,66^ as detailed above.

This may also place the timing of SPA formation into a very specific window in relation to mantle evolution, as it must occur late enough to sufficiently concentrate KREEP and titanium in the upper mantle, but early enough that the mantle had not undergone large-scale downwelling, which would sequester KREEP and Ti in the deep mantle while delivering mafic cumulates to the upper mantle. If IBC downwelling proceeded on a shorter timescale than LMO crystallization^21,23,28^, this could imply that SPA formed while the magma ocean was still partially molten. Could such a lithosphere preserve an impact structure of this immense scale? Perhaps more likely is that IBC/urKREEP downwelling was inefficient, and >30% of this material remained in the upper mantle after LMO solidification and gravitational restructuring^23^.

In either case, it follows that SPA excavation shaped the course of farside mantle evolution. If dense, heat-producing elements were regionally removed from the upper mantle by the impact (and possibly locally induced convection^32,41^) (Fig. 2, bottom panel), this could have rippling effects on farside mantle dynamics and thermal evolution, exacerbated by the unique geophysical environment within the Moon’s largest impact basin. For instance, mantle overturn is thought to be intimately linked to the production of mare basalts and Mg-suite magmas on the nearside. SPA’s effects on the geochemical and geothermal evolution of the farside may explain the unusual volcanic properties of the region, including the relative paucity of mare basalts and the enigmatic volcanic emplacements known as the SPA Compositional Anomaly (SPACA)^58^ and Mons Marguerite (formerly Mafic Mound)^67^.

### Synthesis of remote sensing observations

At face value, the arguments for these different upper mantle compositions seem at odds, and are often treated as such by the lunar remote sensing community. However, this is not necessarily the case. The evolution of the lunar mantle during and after LMO crystallization unfolded over hundreds of millions of years. It is therefore reasonable (or perhaps expected) that impact structures of different sizes, ages, and locations would have exposed upper mantle materials with different compositions. A mantle origin for the olivine observed in Moscoviense does not necessarily contradict a mantle origin for IBC/urKREEP SPA ejecta, and vice versa. Instead, each of these materials is an important piece of a larger lunar evolution puzzle. However, if evidence of a persisting monomineralic upper mantle (dunite or pyroxenite) is discovered, this would suggest that we do not understand the dynamics and timescales of LMO solidification and gravitational restructuring.

While we have heretofore discussed possible surface exposures of mantle materials, crust and volcanic materials are also intimately linked to mantle evolution. A complete understanding of the lunar mantle must therefore be capable of explaining the observed properties of the lunar crust, such as hemispherical differences observed in crustal composition^30^ and volcanic activity^58,67–69^. A holistic model of mantle evolution must rationalize the existence of these diverse materials, whose petrogenesis can be assessed through strategic sample collection and analysis.

## Sampling priorities for future lunar exploration

Because the Apollo and Luna sample return missions covered only a small swath of the lunar surface, the exiting sample collection is grossly incomplete, and contains no known mantle materials. The lunar meteorite collection expands the available sample diversity, but these materials are missing critical spatial and geologic context. While remote sensing observations have greatly increased our knowledge of the composition of the lunar surface, the composition of the mantle can only be approximated from orbit.

Landed missions to the lunar surface are therefore crucial for understanding the formation and evolution of the mantle. It is imperative to integrate remote sensing measurements and sample data to constrain the diversity of rock types and their igneous origin. Seismic measurements of a wider area are also a high priority, revealing the structure, density, and other illuminating properties of the interior. With this knowledge, we can broaden our understanding of planetary differentiation processes, crustal and mantle evolution, and the origin of the Moon.

**Fig. 3:**
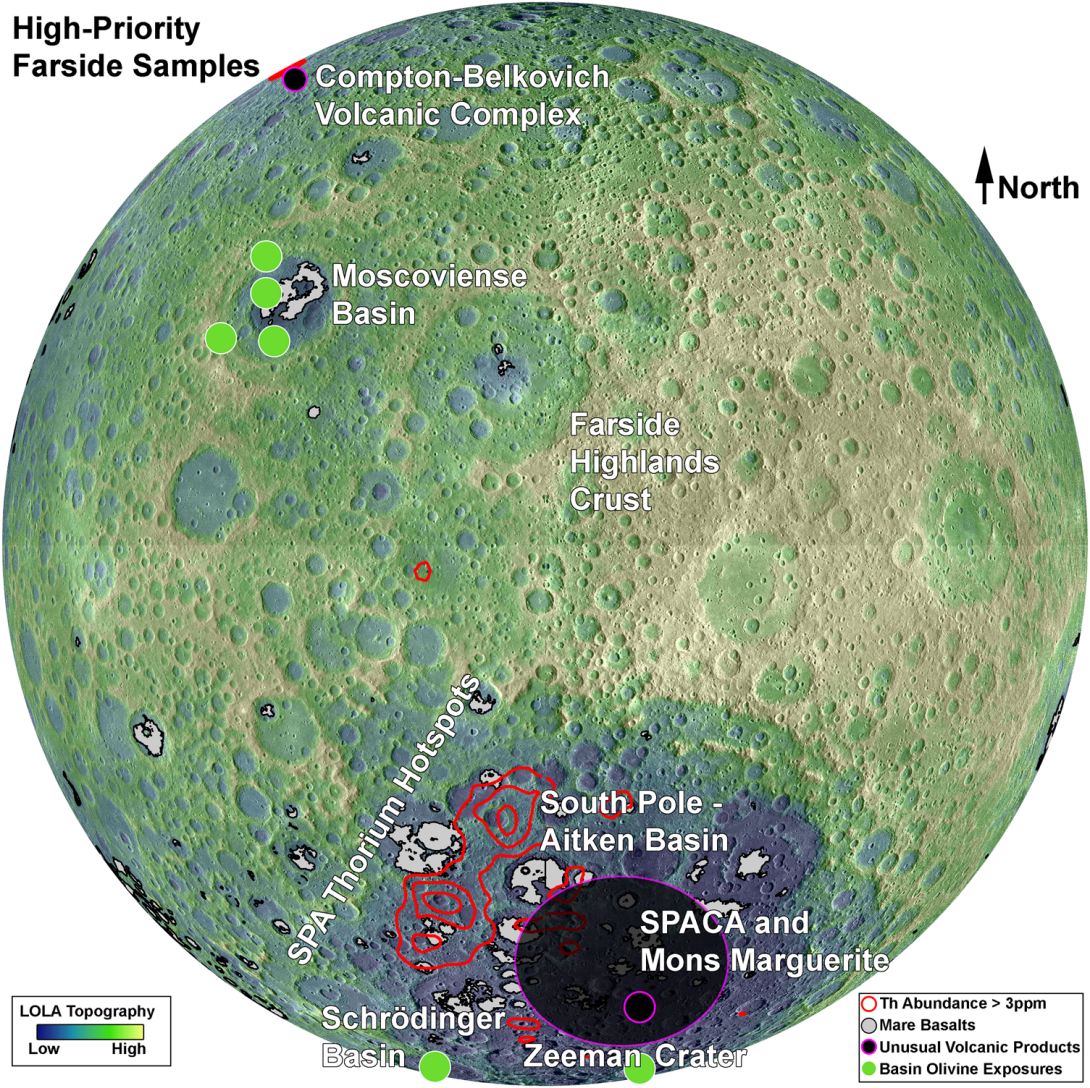
The distribution of high-priority sample targets materials on the currently-unsampled lunar farside. A high concentration of such materials is found within the South Pole – Aitken Basin (SPA) in the southern hemisphere, including the high-Th mantle ejecta and unusual volcanic province referred to as the SPA Compositional Anomaly (SPACA). Crustal materials are widely available across the farside highlands, and low-Ca pyroxenes are observed within many large impact structures. These samples may be available for return to Earth via NASA’s Artemis Program, especially if robotic or other surface assets are able to transport samples from SPA to the Artemis site around the lunar South Pole.

Based on remote sensing observations integrated with an understanding of LMO and mantle overturn models, we suggest five high-priority target lithologies that, if sampled and carefully characterized, could significantly further our understanding of the lunar mantle. The distribution of such materials across the lunar farside is demonstrated in Fig. 3.

### Th-bearing SPA ejecta

This material excavated by the Moon’s largest impact basin bears the compositional signatures (Ca,Fe-rich pyroxenes; elevated Fe, Th, KREEP, and Ti) expected in the upper mantle during the final stages of LMO crystallization^39^. As such, it is the most probable exposure of mantle materials on the lunar surface, and may have been exposed during the final stages of mantle evolution. Sampling these materials is critical for understanding the timing and petrology of magma ocean crystallization.

### Basin olivine exposures

Olivine exposed in several large impact basins may have been excavated from the upper portion of a fully overturned mantle^43,48^. However, remote sensing observations demonstrate significant diversity in olivine abundance and composition within these basins, perhaps more indicative of intrusive Mg-suite magmatism^49^. In either case, determining and characterizing a LMO vs. later magmatic origin for these materials has significant implications for our understanding of mantle restructuring and thermal evolution.

### Basin low-Ca pyroxene exposures

Similarly, low-Ca pyroxene associated with impact basins may also be derived from the lunar mantle. However, there is more potential ambiguity associated with low-Ca pyroxenes, as they are also associated with impact melt, intrusive Mg-suite emplacements, and lower crustal rocks. Of course, sampling a diversity of igneous products will improve our understanding of lunar evolution, even if they are not direct mantle exposures. One question of particular significance to mantle evolution is if pyroxene-bearing mantle materials are monomineralic or part of an assemblage including olivine. Monomineralic pyroxene would indicate that LMO mafic cumulates had not fully mixed or restructured at the time of exposure.

### Farside highlands crust

While the nearside crust was sampled by the Apollo 16 mission, the farside crust differs in several ways. In addition to a greater average thickness, it has been suggested that the farside crust exhibits a more magnesian composition due to asymmetric crustal growth^30^. Sampling the farside highlands crust enables a more detailed, global understanding of how LMO crystallization formed a plagioclase flotation crust, with implications for the bulk composition of the Moon.

### Farside mare basalts and volcanic glasses

Nearside mare basalts were extensively sampled by the Apollo and Luna missions, and form much of the basis for our understanding of the lunar mantle, while geochemistry of lunar glasses has confirmed the presence of IBCs/KREEP in the nearside magma source region^70^. However, farside basalts and glasses differ from nearside basalts in terms of their abundance, composition, and age distribution^69^. Sampling farside mare basalts and glasses will reveal important details about the gravitational restructuring, thermal evolution, and geochemistry of the farside mantle.

### Unusual volcanic materials

While beyond the scope of this article, a number of unusual volcanic emplacements have been observed across the lunar surface, exhibiting anomalous compositions and ages. For instance, the highly silicic Compton-Belkovich volcanic complex^68^ and distinctly non-mare SPA Compositional Anomaly^58^ and Mons Marguerite^67^ reflect unusual igneous processes, while Irregular Mare Patches appear unexpectedly young^71^. These unusual features are not central to reformulating a mantle evolution framework, but their formation must be understood to fully capture the behavior of the lunar mantle through time.

The distribution of these samples on the currently unsampled lunar farside is presented in Fig. 3. Considering the breadth of these samples available in its vicinity, SPA is arguably the highest-priority lunar sample return target. This is not a novel point, as the two most recent Planetary Decadal Surveys listed SPA sample return as one of the highest-priority objectives for advancing solar system science. Several mission concepts have been proposed to NASA in recent years to achieve this objective^72^. Frustratingly, these missions have not been pursued. However, international space agencies have recognized the importance of SPA and have made concerted efforts to explore the basin.

For example, China’s Chang’e-4 mission is currently exploring von Karman crater in SPA. This landing site was selected based on the potential to access material from the lower crust or upper mantle, and the rover, Yutu-2, has a VNIR imaging spectrometer that enables first-order analyses of rock and mineral compositions. Although the interpretation of early results claiming to have identified mantle-derived olivine^73^ has been vigorously challenged^74,75^, the mission is an important milestone in the exploration of the lunar farside.

There is a consensus within the lunar science community that future missions should have the capability to establish absolute ages and measure bulk chemistry and mineralogy of surface materials, either in-situ or by sample return to Earth^76^. NASA’s Artemis program aims to send humans to the lunar South Pole in 2024 and beyond, offering an excellent opportunity for sample return. In particular, a south polar landing site provides access to crustal samples far-removed from the Procellarum KREEP Terrane (PKT). Although not the primary zone of SPA ejecta deposition, it is possible that SPA-derived materials may be present within soils and breccias at the South Pole. Samples from the South Pole thereby offer new insights into the global crustal formation process, and perhaps traces of crust and mantle materials excavated by SPA.

An enhanced science return would be enabled by coordinated robotic rover activity. For instance, a rover could be deployed to central SPA and traverse southward to rendezvous with Artemis astronauts, collecting high-priority science samples along the traverse.

While targeted sample return is essential to fully understand the evolution of the lunar mantle, it is important to consider that, by design, much of the current sample inventory remains unexplored. Over ~80 h of surface exploration, the Apollo astronauts collected 2196 documented samples with a total mass of 381.7 kg^77^. Many samples have been extensively studied by researchers, some nearly to the point of exhaustion^77^. A large portion of the samples, however, remain unstudied. In the next several years, some pristine samples will be opened and analyzed as part of NASA’s Apollo Next Generation Sample Analysis Program. Although these samples are not expected to contain abundant mantle lithologies, researchers will search for mantle fragments within regolith and breccias, and perhaps as xenoliths in basalts.

Understanding the nature and evolution of the lunar mantle has fundamental implications for solar system science, as the Moon offers an accessible laboratory for developing and refining the framework of planetary formation and evolution (See Box 1 for further details). Given the recent advances in our understanding from sample analysis, laboratory experiments, computational models, and remote sensing observations, knowledge of the samples necessary to significantly advance lunar science has been developed, as well as the locations on the lunar surface where those samples are accessible. In a time of renewed interest in lunar exploration by NASA, its commercial partners, and international agencies, new generations of planetary scientists have a clear path towards furthering our understanding of the Moon and beyond.

Box 1:  Evolution  of  rocky  mantles  across  the  solar  system Since the conception of the Lunar Magma Ocean hypothesis, it has been determined that magma oceans are not unique to the Moon. The primary crust of the Earth during the Hadean and the southern highlands of Mars may have been products of magma oceans^78–82^. Magma oceans likely occurred on other rocky bodies during early solar system formation, including Mercury^83^, Io^84^, and differentiated asteroids such as Vesta^85^. However, the terrestrial crust has been repeatedly reprocessed by tectonism, volcanism and other geologic processes, and clear evidence of an early crust been entirely erased. Samples from Vesta, Mars, and other differentiated bodies are limited to meteorites with limited geologic context. Therefore, lunar samples are by far the largest, most accessible collection of primary magma ocean products in the solar system.

Magma oceans across the solar system and beyond differ in their bulk compositions, cooling rates, heat sources, volatile contents, and depths, among other properties. Consequently, each magma ocean is unique in terms of its chemistry, structure, and dynamics. However, deepening our understanding of the lunar magma ocean provides an important baseline through which other bodies can be interpreted. As such, the lunar magma ocean is a keystone for a generalized understanding of magma ocean processes, which may be ubiquitous in terrestrial planet evolution.

For example, gravitational restructuring of the LMO cumulate stratigraphy seems inevitable, but to what extent is this a universal consequence of magma ocean crystallization? How does the range of bulk compositions and crystallization temperatures and pressures affect crystallization sequences, density structures, and viscosity contrasts for other planetary bodies? Do the crystallizing mantles of bodies such as Mars and Earth undergo gravitational restructuring^86, 87^?

Differentiated planetary bodies affected by large impacts may provide further insight into mantle evolution processes across the solar system and beyond. For instance, the ~500 km Rheasilvia Basin on asteroid 4 Vesta is estimated to have exposed abundant upper mantle materials from beneath a relatively thin basaltic crust^88^. However, the dominant mineral observed across Rheasilvia is orthopyroxene, not the more commonly predicted olivine^89^. What were the driving mechanisms affecting the composition structure of Vesta’s mantle, and how do they compare to our understanding of parallel processes affecting the Moon?
